# Deep Brain Stimulation Neuromodulation for the Treatment of Mood Disorders: Obsessive Compulsive Disorder and Treatment Resistant Depression

**DOI:** 10.3389/fpsyt.2021.764776

**Published:** 2022-02-16

**Authors:** Rene Marquez-Franco, Jose Damian Carrillo-Ruiz, Ana Luisa Velasco, Francisco Velasco

**Affiliations:** ^1^Unit for Stereotactic and Functional Neurosurgery, Mexico General Hospital “Dr. Eduardo Liceaga”, Mexico City, Mexico; ^2^Facultad de Ciencias de la Salud, Universidad Anáhuac México, Mexico City, Mexico

**Keywords:** neuromodulation, deep brain stimulation, tractography, mood disorders, treatment resistant depression, obsessive compulsive disorder

## Introduction

Mood disorders like major depressive (MDD) and obsessive compulsive (OCD) disorders affects 300 million people worldwide, and 20–30% are refractory to drug therapy (1). OCD could lead to a lifetime of disabling symptoms while treatment resistant depression (TRD) could lead to suicidal attempts (2, 3). Symptoms common to both disorders include anxiety, sleep disturbances and disruption of selective attention, in contrast to the obsessive thinking and compulsive behavior in OCD, and guilt, concentration problems, sadness and passive behavior in MDD (4).

**Figure 1:**
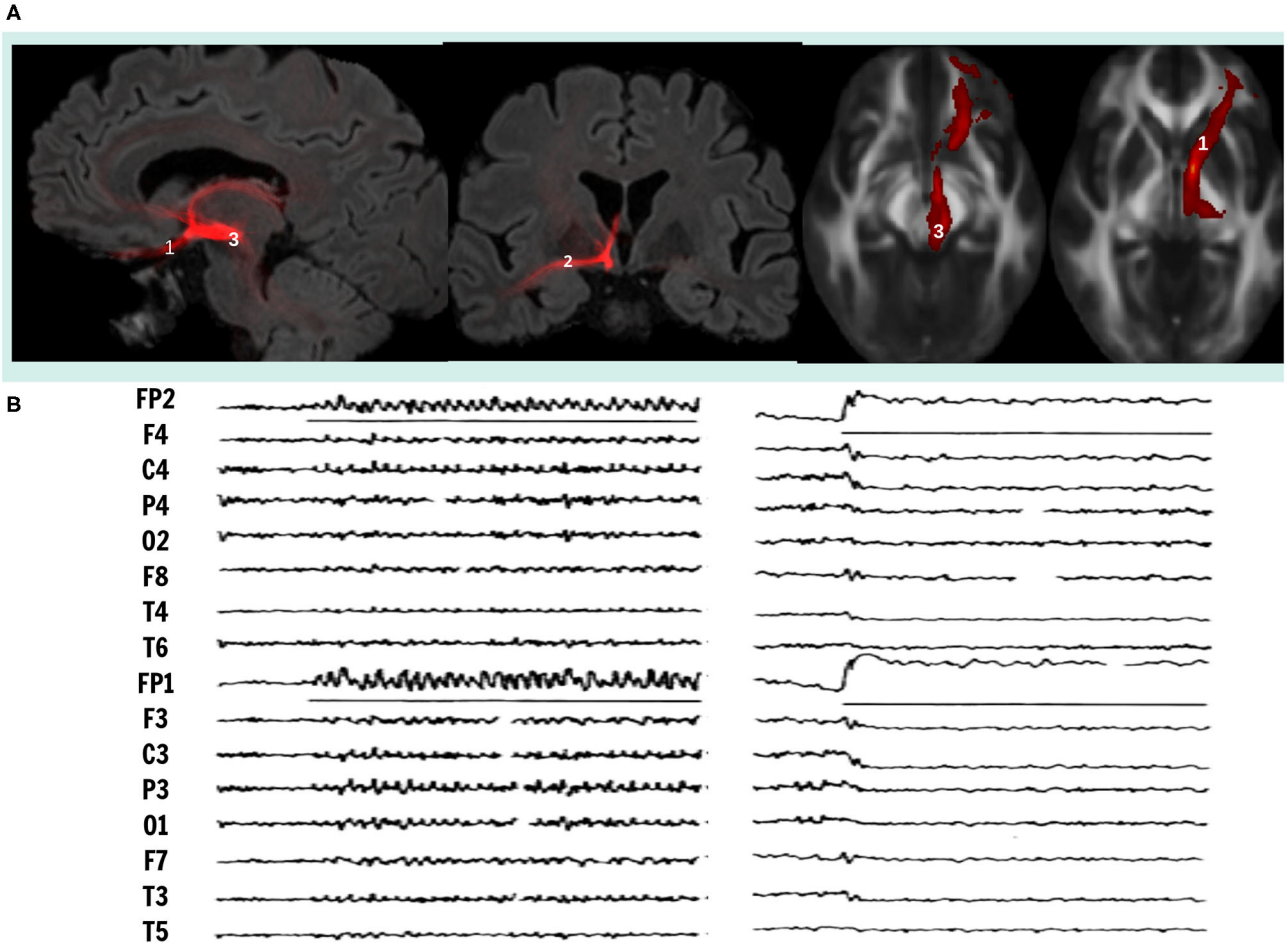
**(A)** Visualization of white matter fiber bundle using probabilistic tractography, that connects orbitofrontal cortex with mesencephalic structures, superimposed to MRI sections in sagittal (left), coronal (center) and axial (right) views. Neuromodulation targets in DBS therapy in research for OCD and TRD, 1, Inferior Thalamic Peduncle; 2, Stria Terminalis; 3, Middle Forebrain Bundle. **(B)** On the left, electrocortical potentials induced recruiting responses by low frequency stimulation (8 Hz, 450 μs, 6.0 V.) and on the right, desynchronization induced by high frequency stimulation (60 Hz, 90 μs, 3.5 V.) on ITP, midline thalamic nuclei and OFC. Adapted from (16).

Deep Brain Stimulation (DBS) has been proposed to treat patient's refractory to drug treatment of these disabling disorders in the same surgical target's network (5). Using probabilistic tractography we visualized that all the proposed targets for the treatment of these disorders have a structural connectivity with the orbitofrontal cortex (OFC), indicating that both pathologies involve dysfunction networks that link OFC with different subcortical and cortical structures (6) (Figure 1). In this report we analyze the physiological, anatomical and biochemical characteristics of the OFC subcortical connections, as well as the mechanisms of DBS, to explain why DBS of the different targets with common structural connectivity may control obsessive-compulsive as well as depressive symptoms, seeking to improve future DBS therapy.

### Neurophysiological Background

Early neurophysiological reports described two systems within the central nervous system, the one linked to transmit single sensory modalities named specific and the other to facilitate the perception of all sensory modalities named non-specific systems (7). Simultaneously, it was reported that punctual low frequency stimulation (LFS) of any midline and intra-laminar thalamic nuclei in cats induced widespread 8 cps monophasic, waxing and waning cortical potentials, named recruiting responses (8), while high frequency stimulation (HFS) in the same locus induced generalized cortical desynchronization (9). LFS of OFC non-specific thalamic nuclei is accompanied by sleepy and inattentive behavior while HFS awaked the animals and increased selective attention (10). Two decades later it was reported that LFS of OFC and their fibers connecting with non-specific thalamic nuclei (NST) in cats induced the same generalized cortical recruiting responses, accompanied by sleeping and inattentive behavior (11).

In contrast, lesions of the OFC plus the ventral caudate nucleus render the cat hyperactive, with compulsive walking and eating and without orienting reaction. Lesions restricted to mesial OFC, or cooling of fibers connecting OFC with NST in the inferior thalamic peduncle (ITP) blocked recruiting responses and induced methodic walking, with animals unable to switch directions in the presence of an obstacle and remaining pawing the cage, they persevered following a visual stimulus regardless its information content (12, 13). In humans, sub caudate leucotomies interrupting OFC pathways and ventral caudate nucleus induce compulsive hyperactivity and impaired attention (14). In contrast, lesions circumscribed to the posterior mesial OFC (area 13) were effective to improve medical treatment resistant depression (TRD) in a large series (15). LFS induced recruiting responses and HFS cortical desynchronization induced by centromedian thalamic nuclei in patients with epilepsy (16) and inferior thalamic peduncle in patients with TRD and OCD, have been used to confirm the correct position of DBS electrodes in non-specific system. However, LFS to induce recruiting responses requires 8–15 V., 450 μs and 8 cps stimulation, while HFS inducing cortical desynchronization requires 2–5 V, 90 μsand > 60Hz (17) (Figure 1).

### Anatomical Background

Pathways between OFC and NST, cortical (pre-piriform, anterior ventral frontal, cingulate and sugenual limbic cortices) and basal ganglia (caudate and putamen) are bidirectional and their inactivation produce similar effects on recruiting responses, spindle burst and motor behavior (18–21). Sub cortical connections include ventral striatum, hypothalamus, Nucleus Acumbens (NAcc), substantia nigra, and ventral tegmental area. Dysfunction of these circuit results in disinhibition, impulsiveness and emotional lability with and low tolerance to change strategies and impaired performance in a task related to reward and punishment (22). Mesencephalic connections with OFC are bilateral (23) most likely through the Middle Forebrain Bundle (MFB), unilateral lesions in these connections near the mesencephalon renders the patient inattentive to contralateral limbs, indicating that the connections are involved in the process of selective attention (24, 25). Lesions in OFC, caudate nucleus and cingulate gyrus in humans and animals indicate that these anatomical areas are implicated in the evaluation of stimulus significance as positive (rewarding) and negative (punishing) and critical for habit learning and acquisition of stereotyped behavior (26). OFC lesions in humans derive to difficult decision making through estimating the positive or negative consequence of particular actions (27), resulting in abnormalities in reward expectations and preferences (28). Ventromedial OFC lesions decrease prediction on winning in gambling tasks and its metabolic activity is proportional to the uncertainty of outcomes (29). Restricted chemical lesions of rostral cingulate gyrus render the monkeys incapable in selecting actions based on reward associated paradigms (29), OFC and cingulate gyrus influence emotional value of stimuli and selects the behavioral response based in previous experiences. Basal ganglia and NST mediate patterns generated in brain stem circuits, spinal cord (motor patterns) and those generated in the cerebral cortex (cognitive patterns). In OCD, the cortical-basal ganglia system is unable to shift from one priority to another and remains locked to a specific behavior (2, 5, 26).

### Neurochemical Substrates of MDD and OCD

Paroxetine, a serotonin re-uptake inhibitor, improves OCD and MDD, which provides evidence of relationship between serotoninergic dysfunction in these disorders. In an experimental model of perseverative behavior induced by chemical OFC lesions in rats, associated to a paradigm of luminous signal indicating food rewarding by pressing a lever (30), Thereafter, luminous signal was attenuated and pressing the lever was no longer associated with rewarding. Lesioned animals were compared with intact rats as far as the excessive lever pressing (ELP) after luminous attenuation. While intact animals learned not to respond to attenuated luminous signal after one or two trials, Lesioned animals persevered in ELP. This perseverative behavior was prevented by the administration of Paroxetine. At the end [3H] imipramine binding was determined in striatal cell membranes as evidence of adrenergic transporter, since the striatal cell membranes are devoid of adrenergic transporters, increase in imipramine binding was due to increase of serotoninergic transporters in lesioned animals. In other experiments, restricted chemical lesions in rostral cingulum gyrus render monkeys incapable of selecting actions on reward association paradigms (31), while ventromedial OFC lesions restrict the prediction of winning responses in gambling tasks and metabolic activity in OFC was proportional to uncertainty of outcomes (29).

Several neurotransmitters, neuropeptides and hormones are involved in the physiopathology of MDD (1). Low levels of norepinephrine correlate with depression and high levels correlate with mania (32) and α2-noradrenergic presynaptic and β-adrenergic receptors were consistently found increased in the frontal lobes of depressed individuals that committed suicide (33). This has been interpreted as increase in receptor activity in response to decreased release of norepinephrine in depressed patients. Abnormal metabolism of 5- HT accounts for down regulation of β-adrenergic receptors and desensitization of 5-HT1A auto receptors in depressed patients (34). Dysregulation of 5-HT metabolism in depression correlates with over activity of OFC. Therefore, serotonin reuptake inhibitors increase serotonin availability and are potent antidepressants. Even more, a low-triptophan diet decreases serotonin synthesis and may lead to a relapse of depression (35). All these findings have linked the MDD with OFC cortico-basal ganglia network mediated by serotonin and adrenergic systems (36).

### Imaging Studies

Increased metabolic activity in OFC is present in patients with MDD that normalizes when the symptoms are controlled by medication, as demonstrated in ^18^FDG-PETstudies (37). In OCD increased metabolism has been reported also in OFC, and anterior dorsolateral prefrontal cortex, ventral caudate nucleus and medial thalamus (38). Provocative maneuvers that increase OCD symptoms increase regional cerebral blood flow (rCBF) and metabolic activity in right lateral dorsal-frontal cortex, while improvement induced by chlor-imipramine correlated with the largest metabolic change on right anterior orbitofrontal cortex. Moreover, left anterior OFC activation correlated positively with symptom intensity, while left posterior OFC activation correlated negatively with symptom intensity (38). In a subsequent study, regional metabolic activity in OFC was carried out dividing OFC in anterior-lateral and posterior-medial segments, with different cytoarchitecture and patterns of connectivity. Metabolic PET studies in OCD patients revealed that posterior medial OFC showed maximal hyperactivity that decreased with the administration of Paroxetine, probably mediating OCD symptoms. In contrast, anterior-lateral OFC increased metabolic activity when OCD symptoms improved. Posterior medial OFC connects mainly with cortical and subcortical limbic regions and probably mediates emotional symptoms, while anterior lateral connects with thalamic and associative pre-frontal cortices mediating cognitive decisions (39).

### Neuromodulation for the Treatment of OCD and TRD

Electrical stimulation generated through an implantable pulse generator and connected to multi-contact electrodes implanted into the brain, known as DBS, was proposed for the first time in 1999 to treat OCD patients, using the anterior limb of the internal capsule (ALIC) (40), that had proven to be an effective target for radiofrequency and radiosurgery lesions (41). Neuromodulation using DBS for the treatment of intractable mental illness are based on a neural network theory in which it is postulated that a certain set of structurally and functionally connected regions of the brain work together to maintain normal regulation of mood (42). DBS applied in the ITP, identified by trans-operative recruiting responses and cortical desynchronization resulted in an immediate improvement of a TRD (17, 43). Later on ITP-DBS was proposed to treat OCD symptoms with a favorable results (44, 45), however, in contrast to the immediate response obtained in MDD, in OCD the favorable effect appeared weeks to months after the onset of DBS (see Supplementary Videos). Other targets for treating OCD include the basal nucleus of the stria terminalis (BNST) (46), the NAcc, and the superior lateral branch of the middle forebrain bundle (slMFB) (6, 22).

Beyond the selected target to treat OCD and TRD there are questions that need to be resolved for improving DBS in the treatment of mental illness:

1. **Stimulation Parameters**: DBS for the treatment of TRD and OCD in different targets has been performed with HFS (>60 Hz, ± 90 μs and 3.0 V.) derived from experience of DBS in movement disorders. As mentioned before, HFS increases alertness and hyperkinesia when applied to non-specific reticulo-thalamic system, while LFS (8 cps, 450 μs, >5.0 V.) is required to induce cortical synchronization and hipokynesia. Lesions and HFS applied in the white matter fiber bundle between red nucleus and sub thalamic nucleus, within the prelemniscal radiations (Raprl) for the treatment of Parkinson's disease on a white matter fiber bundle connecting mesencephalon with the OFC, reduced hipokynesia (47) and induce a highly significant decrease in the metabolic activity of OFC in both sides (48). This white matter fiber bundle has been identified as the superior lateral branch of the middle forebrain bundle (slMFB) and reported effective in controlling TRD and OCD (6). Therefore, DBS on ALIC, ITP and MFB are effective in releasing symptoms of both disorders.
  Although the cellular mechanisms of HFS and LFS DBS are complex (49), LFS in some targets is more effective in controlling specific symptoms, as in the case of peduncle-pontine nucleus for control of gait (50) or the cortical stimulation to control pain (51). In a model of perseverative behavior induced by administration of (8) OH-DPTA, LFS of midline and reticular nuclei induced significant reductions of perseverative responses (52). Perhaps the best indication to test the effects of HFS and LFS in mental disorders would be the cases such as of MFB-DBS in a patient with bipolar TRD and comorbid OCD reported (53).
2. **Target Size and Location**: DBS systems are designed to stimulate discrete areas around the active contacts, therefore, the target's volume must be small, precisely localized by imaging and electrophysiological methods and away from other structures that could induce undesirable effects. As proposed by Spiegel and Wycis in the 1963, conforming lesions in cerebral nuclei is complicated in view of their shape and size, in contrast, white matter fiber bundles connecting nuclei may represent a better volume for lesioning or stimulation (54). With the advances in probabilistic and deterministic tractography, the stereotactic location of different white matter fiber bundles can be precisely identified, moreover, the point of maximal proximity of the fiber bundles can be determined and would represent the optimum site for DBS or lesioning for the selected target.
3. **Trans-Operative Confirmation of the Target:** Recording electrophysiological signals through microelectrodes helps to confirm cellular activity of nuclei. Electrophysiological responses to LFA and HFS helps to optimize the location of fiber bundles in several targets (16, 17). Trans-operative HFS stimulation through DBS electrodes alert the proximity of nuclei and fibers that may induce undesirable effects when conforming DBS volume.

## Conclusions

Reticular-thalamic-cortical system mediates contrasting functions like sleep-wakefulness cycles, attentive and non-attentive conditions, approaching or avoiding behaviors. DBS to treat TRD and refractory OCD should focus in: 1. Determining by Diffuse Weighted Imaging (DWI) if posterior medial and anterolateral OFC are connected with cortical and subcortical structures by different fiber tracts, running in parallel and mediating depressive vs. obsessive compulsive behaviors. 2. If in the same fiber tract low frequency high amplitude improves OCD and high frequency low amplitude improves TRD, in the same way that non-specific thalamic nuclei low frequency high amplitude stimulation induces sleep and high frequency low amplitude induces awakening. Optimization of therapy will include probabilistic and/or deterministic tractography guiding electrode implantation, high amplitude low frequency vs. low amplitude stimulation and size and location of the target, away from structures where DBS could induce undesirable side effects.

## Author Contributions

FV: original idea, manuscript writing, and surgeon in charge of the mood disorders (OCD and TRD) patients that underwent to surgery. RM-F: probabilistic tractography analysis and manuscript writing. JC-R: surgeon in charge of the mood disorders (OCD and TRD) patients that underwent to surgery. AV: neurologic evaluation and neurophysiological recordings of patients that underwent to surgery for mood disorders (OCD and TRD). All authors contributed to the article and approved the submitted version.

## Funding

RM-F is a doctoral student from the Programa de Doctorado en Ciencias Biomédicas, Universidad Nacional Autónoma de México (UNAM) and has received from the Consejo Nacional de Humanidades, Ciencias y Tecnologías (CONAHCYT) the fellowship number 939081.

## Conflict of Interest

The authors declare that the research was conducted in the absence of any commercial or financial relationships that could be construed as a potential conflict of interest.

## Publisher's Note

All claims expressed in this article are solely those of the authors and do not necessarily represent those of their affiliated organizations, or those of the publisher, the editors and the reviewers. Any product that may be evaluated in this article, or claim that may be made by its manufacturer, is not guaranteed or endorsed by the publisher.

## Supplementary Material

Supplementary Videos Patient with TRD responding question of Zung's scale for Major Depressive Disorder, **(A)** Pre-operative condition, **(B)** Post-operative condition 2 days after onset of DBS on the ITP. **(C)** Pre-operative condition of a patient with OCD, answering questions of Y-BOCS, during an interview with the neuropsychologist. **(D)** Same patient interviewed 3 months after the onset of DBS in the ITP. Patient with TRD responding question of Zung's scale for Major Depressive Disorder, (1) Pre-Surgery, Zung Evaluation for Major Depressive Disorder: https://drive.google.com/file/d/1hFQWGZ5yJUDKoiZ3CcM_9Hi3T-8pz216/view. (2) Post-operative condition 2 days after onset of DBS on the ITP. Post-Surgery, Zung Evaluation for Major Depressive Disorder: https://drive.google.com/file/d/1uWQStQbiXLkcBmETHha6BX-PvR9oD-7y/view. *Pre-*operative condition of a patient with OCD, answering questions of Y-BOCS, during an interview with the neuropsychologist. (1) Pre-Surgery: https://drive.google.com/file/d/1WqiXRzt48LiGQcRAWiTKOzvM-VHzO3R3/view. (2) Post-Surgery, same patient interviewed 3 months after the onset of DBS in the ITP: https://drive.google.com/file/d/1hGfouaVJK0a67icPegT-rEGvIfDGBffp/view.
