# Corrigendum: Deep brain stimulation neuromodulation for the treatment of mood disorders: obsessive compulsive disorder and treatment resistant depression

**DOI:** 10.3389/fpsyt.2023.1310446

**Published:** 2023-11-30

**Authors:** Rene Marquez-Franco, Jose Damian Carrillo-Ruiz, Ana Luisa Velasco, Francisco Velasco

**Affiliations:** ^1^Unit for Stereotactic and Functional Neurosurgery, Mexico General Hospital “Dr. Eduardo Liceaga”, Mexico City, Mexico; ^2^Facultad de Ciencias de la Salud, Universidad Anáhuac México, Mexico City, Mexico

**Keywords:** neuromodulation, deep brain stimulation, tractography, mood disorders, treatment resistant depression, obsessive compulsive disorder

In the published article, there was an error in the **Funding** statement.

The previous statement was: “RM-F received fellowship 939081 from the National Council of Science and Technology, Mexico (Consejo Nacional de Ciencia y Tecnología, CONACYT).”

The correct **Funding** statement appears below:

